# Extensive Left Ventricular Thrombosis with Concomitant Pulmonary Embolism

**DOI:** 10.3390/clinpract11020043

**Published:** 2021-05-18

**Authors:** Annamária Magdás, Cristian Podoleanu, Attila Frigy

**Affiliations:** 1 Department of Internal Medicine, County Clinical Hospital Mureș, 1 Gheorghe Marinescu Street, 540103 Târgu Mureș, Romania; 2 George Emil Palade University of Medicine, Pharmacy, Science and Technology of Târgu-Mureș, 38 Gheorghe Marinescu Street, 540142 Târgu Mureș, Romania; cristian.podoleanu@umfst.ro (C.P.); attila.frigy@umfst.ro (A.F.); 3 Department of Cardiology, County Clinical Hospital Mureș, 1 Gheorghe Marinescu Street, 540103 Târgu Mureș, Romania

**Keywords:** ventricle, thrombi, pulmonary embolism, 3D echocardiography

## Abstract

A 57-year-old non-obese female patient with a history of heavy smoking, chronic obstructive pulmonary disease and hypertension was admitted to the hospital as an emergency for acute hemoptysis and signs of congestive heart failure. To assess the source of hemoptysis, computed tomography (CT) pulmonary angiography was performed, which confirmed a bilateral pulmonary embolism of the apical branches. The routinely performed transthoracic echocardiography (TTE) revealed an enlarged left ventricle with severely reduced ejection fraction (EF = 25%) due to global hypokinesia and multiple, mobile, echogenic masses. To increase the diagnostic accuracy, real-time three-dimensional (Live 3D) imaging of the masses was added which described multiple left ventricular (LV) thrombi. Successful resolution of intraventricular thrombi was noticed after treatment with oral anticoagulant therapy (acenocumarol), despite the lack of regular INR control.

## 1. Introduction

The formation of left ventricular thrombi (LVT) is related mainly to slow flow conditions caused by global or segmental systolic motion anomalies of the left ventricular walls. This can occur in the setting of acute or chronic myocardial ischemia (acute myocardial infarction, hibernated myocardium, myocardial scarring) or non-ischemic dilated cardiomyopathy [1]. In the case of LVT, the pathophysiologic background is represented by Virchow’s triad, which includes blood stasis, hypercoagulability and endocardial injury [2]. Since the majority of reported cases were secondary to acute myocardial infarction, LVT are associated with high mortality, and so early recognition and prompt therapy is mandatory. In this context, transthoracic echocardiography (TTE) is the most valuable tool in clinical practice for detecting LVT in patients with high risk for intracavitary thrombosis–heart failure with decreased left ventricular ejection fraction and/or important segmental (mainly apical) wall motion anomalies. In the following, we present a particular case of simultaneous pulmonary embolism and extensive LVT in a patient with dilated cardiomyopathy and advanced heart failure.

## 2. Case Report

**Figure 1:**
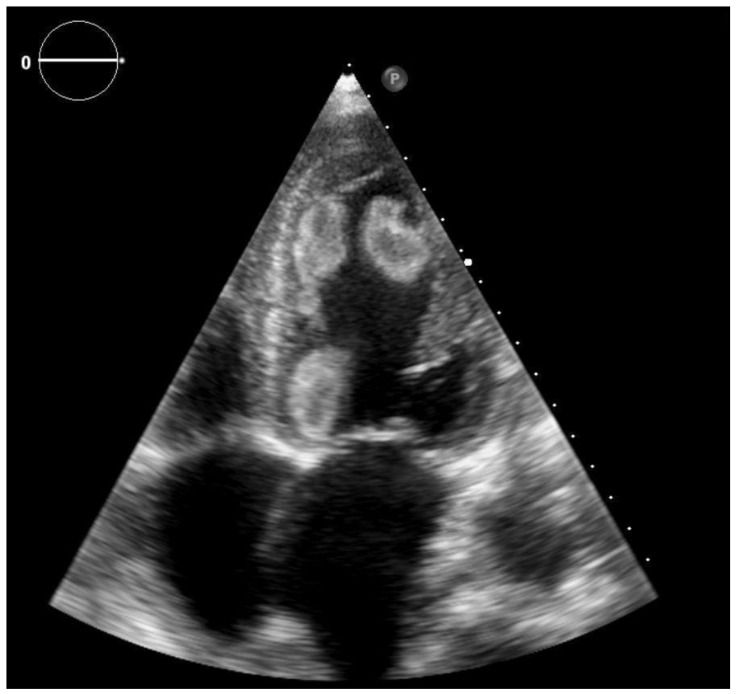
2D transthoracic echocardiography, apical four chamber view—the left ventricular cavity is filled by multiple thrombi: apical-septal (AS), -lateral (AL) and basal-infero-septal (BIS).

**Figure 2:**
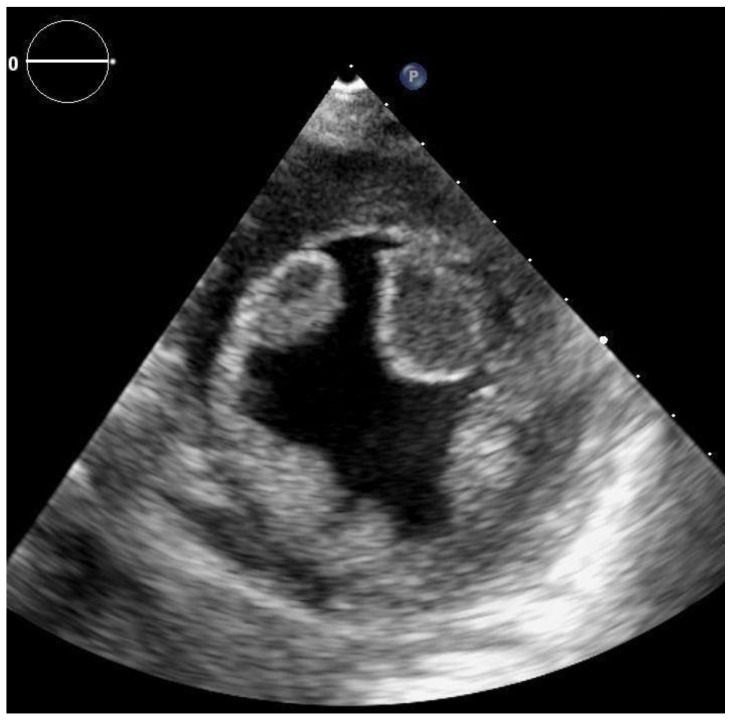
2D transthoracic echocardiography, parasternal short axis view at the level of papillary muscles—visualization of apical-septal (AS) and -lateral (AL) thrombi in the anterior part of the left ventricle.

A 57-year-old female was admitted to the hospital as an emergency for acute hemoptysis and dyspnea. The patient was non-obese, with a history of heavy smoking, chronic obstructive pulmonary disease and hypertension, and at the time of admission presented signs of congestive heart failure. Family history was negative for any thrombotic disease. Regarding past medical history the patient had no previous miscarriage, neither deep vein thrombosis, nor recent prolonged bed rest or hospital admissions. The only prothrombotic factor that we could identify was smoking. Due to the high clinical suspicion, computed tomography (CT) pulmonary angiography was performed, which confirmed a bilateral pulmonary embolism of the apical branches. On the electrocardiogram, sinus rhythm was detected with multiple supraventricular premature beats, but without significant morphological (QRS, ST-T) changes. The routinely performed TTE with a Philips Epiq 7 ultrasound system revealed an enlarged left ventricle with severely reduced ejection fraction (EF = 25%) due to global hypokinesia and multiple, mobile, echogenic masses (for details see Figure 1 and Figure 2). In order to better characterize LV masses, real-time three-dimensional (Live 3D) imaging was added. Since these mobile masses belonged to areas of abnormal wall motion, they were defined as LV thrombi. The apical-septal one measured 31.9 × 13.7 mm, the other located apical-laterally measured 24.5 × 16.5 mm and the basal-infero-septal one measured 28.2 × 20.3 mm.

**Figure 3:**
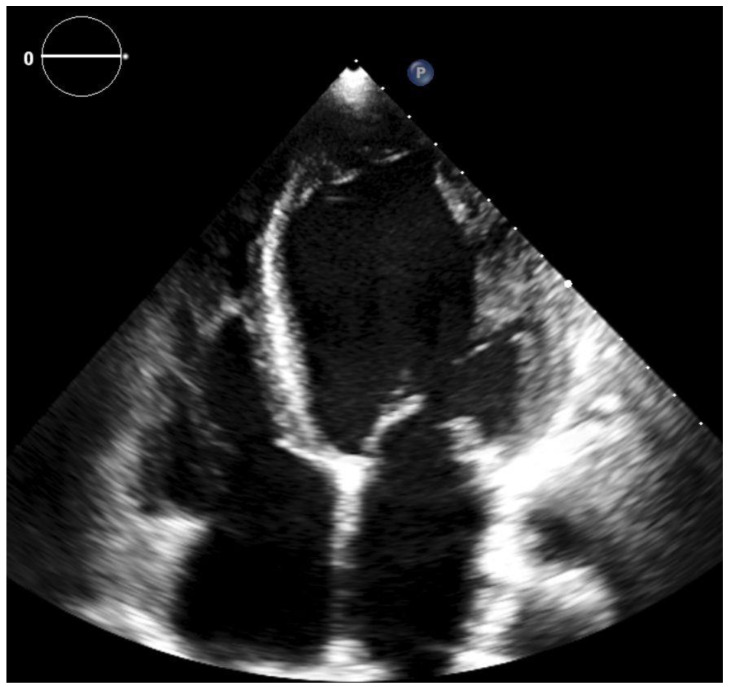
2D transthoracic echocardiography, apical four chamber view (second examination)—no thrombi are visible in the left ventricular cavity.

To find out the source of pulmonary embolism and as primary screening for malignancies, venous and abdominal ultrasound examinations were performed. Venous duplex Doppler examination of the lower limbs excluded the presence of deep vein thrombosis. Embolic or ischemic etiology was not ruled out by angiography; however, the patient had no history of angina or myocardial infarction, and there were no regional wall motion anomalies on echocardiography suggesting coronary disease. We considered the cardiomyopathy as having non-ischemic etiology. During the hospital stay, the patient developed a transient alteration of neurological status with dysarthria and signs of central facial palsy. Cerebral CT was performed, which ruled out significant changes, and the neurological signs regressed completely in 36 h. From the beginning of the hospital stay, therapeutic anticoagulation was started with enoxaparin, and continued later with acenocumarol. The further in-hospital clinical course of the patient was uneventful, and she was discharged after 7 days, on oral anticoagulation with acenocumarol. Since our patient was hemodynamically stable, there was no indication for systemic thrombolysis. Before initiating anticoagulant treatment the main coagulation parameters of the patient were: erythrocytes 5.27 × 106/µL, hemoglobin 15.98 g/dL, hematocrit 50.20%, platelet 259.10 × 103/µL and D-dimers were positive, INR 1.19. Unfortunately, because of poor social conditions, the patient was lost to follow-up, and it was not possible to track her INR values or to perform thrombophilia testing after 6 months of anticoagulant treatment. Eight months after the index event the patient was re-admitted for a respiratory failure and signs of acute heart failure. The repeated TTE revealed an EF of 15% and total resolution of the LVT (see Figure 3).

## 3. Discussion

Intracardiac thrombus formation is a potentially severe complication related to cardiac conditions such as ischemic and non-ischemic cardiomyopathies (with or without manifest heart failure), valvular diseases and atrial fibrillation [3,4]. In the pathophysiology of LVT development the elements of Virchow’s triad play the main role: (1) wall motion abnormalities causing slow flow conditions and blood stasis, (2) the presence of local and systemic proinflammatory factors provoking endocardial lesions and (3) hypercoagulability [5,6]. A recent study found that the most frequent condition associated with LVT was heart failure, with 38% of the subjects being newly diagnosed cases, and myocardial infarction being the second most common promoter. Among these subjects the in-hospital and the one-year mortality rate was 7.8 and 13%, respectively [7]. Intracavitary thrombosis appears in advanced stages of cardiomyopathies and the risk seems to be higher in the non-ischemic forms [8]. Previous studies revealed an incidence of 19.2% of LVT formation after an acute ST-elevation myocardial infarction with ejection fraction <50% [9].

Cerebral infarction, pulmonary embolism and multi-organ (systemic) embolization were reported as possible complications of LVT [10]. In untreated LVT the risk of embolization is estimated to be around 10–15% in the first three months, while in the case of protrusive, mobile thrombi this percentage can rise up to around 60% [4,11]. Although, under efficient anticoagulation therapy, resolution of thrombi is achieved in a median period of 63 days, it has been shown that late resolution—longer than 1 month—of an apical thrombus is associated with poor long-term survival [12].

Our case, a patient with heart failure due to dilated cardiomyopathy with severely reduced ejection fraction of the left ventricle despite sinus rhythm, developed apical-septal and -lateral, as well as basal-infero-septal thrombi of the left ventricle with concomitant pulmonary embolism and a transient ischemic attack as a manifestation of a cardioembolism. The clinical context and the typical aspect on echocardiography conferred the diagnosis of extensive left ventricular thrombosis. If the echocardiographic diagnosis of intracavitary masses is suspected not to be accurate, cardiac magnetic resonance imaging using gadolinium contrast, which is considered the gold standard of diagnosis, can be performed, but this imaging method was not required for the diagnosis in our case. In the absence of malignancies, beyond local factors, hereditary thrombophilia was suspected as a possible co-factor for the association of the observed extensive LVT and concomitant pulmonary embolism. Regarding the treatment options, although in the case of mobile, protrusive thrombi successful surgical extractions were reported, prompt initiation of anticoagulation therapy is the most widely available and first-line option in the prevention of systemic embolization [11,13]. In our case, despite not having the INR values tracked, the patient had, presumably, an efficient long-term anticoagulation with the vitamin K antagonist, acenocumarol (INR at the second admission was 3.6), which was responsible for the resolution of LVT. Although prospective randomized controlled trials are still missing, direct oral anticoagulants (DOACs) could represent a valid therapeutic option for the treatment of LVT [14].

## 4. Conclusions

We described the case of a patient with dilated cardiomyopathy with very low ejection fraction, in sinus rhythm, who had multiple, extensive LVT associated with concomitant pulmonary embolism and a transient ischemic attack of cardioembolic origin. Our case draws attention to the multiple and complex manifestations of the local and systemic thrombogenic milieu, which can develop in the setting of heart failure. Systemic thrombolytic therapy in our patient, in the case of a high-risk pulmonary embolism, could have had a deleterious effect, bearing an important risk of systemic embolism. Mobile, multiple and protrusive LVT seen on echocardiography represent a high risk for systemic embolization, therefore an efficient and long-term anticoagulant therapy should be introduced without delay. Transthoracic echocardiographic examination applying three-dimensional imaging has an increased diagnostic sensitivity helping to determine with more accuracy the nature of an intracardiac mass.

## References

[1] Jiang X.Y., Jing L.D., Jia Y.H. (2015). Clinical characteristics and risk factors of left ventricular thrombus after acute myocardial infarc-tion: A matched case-control study. Chin. Med..

[2] Delewi R., Zijlstra F., Piek J. (2012). Left ventricular thrombus formation after acute myocardial infarction. Heart.

[3] Ye F., Silverstein B.V., Khuddus M.A., Bray C.L., Lee A.C. (2018). Giant Left Ventricular Thrombus in a Patient with Acute Ischemic Stroke: A Case Report and Minireview. Case Rep. Cardiol..

[4] Pol D., Perera P., Zaman S. (2019). Multiorgan embolization of a left ventricular thrombus. BMJ Case Rep..

[5] Garg P., Van Der Geest R.J., Swoboda P.P., Crandon S., Fent G.J., Foley J.R., Dobson L.E., Al Musa T., Onciul S., Vijayan S. (2019). Left ventricular thrombus formation in myocardial infarction is associated with altered left ventricular blood flow energetics. Eur. Heart J. Cardiovasc. Imaging.

[6] Cevik C., Shah N., Wilson J.M., Stainback R.F. (2012). Multiple left ventricular thrombi in a patient with left ventricular noncom-paction. Tex. Heart Inst. J..

[7] McCarthy C.P., Murphy S., Venkateswaran R.V., Singh A., Chang L.L., Joice M.G., Rivero J.M., Vaduganathan M., Januzzi J.L., Bhatt D.L. (2019). Left ventricular thrombus. contemporary etiologies, treatment strategies, and outcomes. J. Am. Coll. Cardiol..

[8] Habash F., Vallurupalli S. (2017). Challenges in management of left ventricular thrombus. Ther. Adv. Cardiovasc. Dis..

[9] Bulluck H., Chan M.H.H., Paradies V., Yellon R.L., Ho H.H., Chan M.Y., Chin C.W.L., Tan J.W., Hausenloy D.J. (2018). Incidence and predictors of left ventricular thrombus by cardiovascular magnetic resonance in acute ST-segment elevation myocardial infarction treated by primary percutaneous coronary intervention: A meta-analysis. J. Cardiovasc. Magn. Reson..

[10] Mine T., Sato I., Miyake H. (2014). Multiple left ventricular thrombi in a patient with dilated cardiomyopathy and cerebral infarction: A case report. J. Med. Case Rep..

[11] Rodrigues P., Sousa M.J., Caiado L., Cabral S., Meireles A., Santos M., Palma P., Torres S. (2016). Intracardiac thrombus and Murphy’s law: Reflections on a clinical dilemma. Rev. Port. Cardiol..

[12] Oh J.K., Park J.-H., Lee J.-H., Kim J., Seong I.-W. (2019). Shape and Mobility of a Left Ventricular Thrombus Are Predictors of Thrombus Resolution. Korean Circ. J..

[13] Gliga M., Gomotârceanu A., Podeanu D., Dogaru G. (2012). Multiple renal infarctions due to thromboembolism. Importance of ultrasound in diagnosis. Case report. Med. Ultrason..

[14] Tomasoni D., Sciatti E., Bonelli A., Vizzardi E., Metra M. (2020). Direct oral anticoagulants for the treatment of left ventricular throm-bus-a new indication? A meta-summary of case reports DOACs in left ventricular thrombosis. J. Cardiovasc. Pharmacol..

